# A Prospective Multicenter Study to Evaluate the Safety and Efficacy of the Topical Application of MYOWNN™, an Autologous Growth Factor Concentrate (AGFC) Serum, in Anti-Aging

**DOI:** 10.7759/cureus.25190

**Published:** 2022-05-21

**Authors:** B. S. Chandrashekar, Kalpana Sarangi, Mushtaque A Mastim, Ashima Bhatia, Manishkumar Shah, Vijay Sharma, Mugdha Gupta, Ranjeet Gutte, Anuka Sharma, Ulka Sakhalkar

**Affiliations:** 1 Dermatology, Cutis Academy of cutaneous sciences, Bengaluru, IND; 2 Dermatology, DKS Clinique, Mumbai, IND; 3 Global Clinical Development, Wockhardt Ltd., Mumbai, IND; 4 Stem Cell Research, Wockhardt Ltd., Mumbai, IND

**Keywords:** wrinkle severity rating scale, growth factor, wrinkles, antiaging, rejuvenation

## Abstract

Background

Growth factors from platelets have been emerging as a revolutionary treatment with the ability to induce cell growth in the skin, which results in retarding and reversing the aging process. Platelet-rich plasma (PRP) allows for greater release of growth factors and biologically active proteins, which in turn activates the cascade of stimulation of neoangiogenesis and collagen production. PRP is used in anti-aging and facial skin rejuvenation in the form of dermal injections and topical application during micro-needling. This study was conducted to assess the safety and efficacy of a topically applied face serum, MYOWNN™ (Wockhardt Ltd., Mumbai, India). MYOWNN™ is an autologous growth factor concentrate that has been made into a topical face serum.

Methods

Male and female subjects in the age group between 30 and 55 years (both inclusive) with Fitzpatrick skin type III-V who had not taken any oral or topical treatments for at least four weeks before and any platelet-rich plasma (PRP) based facial treatment (injections) at least six months before the study entry were included. MYOWNN™ serum was applied on the face once daily at night, approximately 30 minutes before sleeping preferably, for a total duration of five months. Six parameters, i.e. spots, pores, wrinkles, texture, moisture, and pigmentation, were evaluated at regular intervals with Visage-LS (dermaindia®, Tamil Nadu, India), a face analysis system that gives the live status of these six parameters and is an advanced live status skin detection equipment together with shooting, analyzing, and displaying functions, as well as the subjective analysis, was performed by subjects and physicians using different globally accepted scales like physician’s global aesthetic improvement scale (PGAIS), subject’s global aesthetic improvement scale (SGAIS), subject satisfaction score (SSS), and wrinkle severity rating scale (WSRS). For analysis, a mixed model for repeated measures was used. The model had change from baseline as the dependent variable visit as a factor and baseline assessment result as a covariate. All primary and secondary efficacy endpoints were analyzed using Modified Intent-to-Treat (mITT) populations.

Results

Improvement in an average of six anti-aging parameters was observed as early as three months while statistically significant improvement was observed by the end of five months of application. A statistically significant improvement in wrinkles was observed by the end of three months of the application itself. There were no product-related adverse events reported.

Conclusions

Five months of application of MYOWNN™ serum showed a statistically significant improvement in an average of six parameters of anti-aging and face rejuvenation with a p-value of 0.0150 (<5% level of significance (i.e. 0.05) and was also well-tolerated.

## Introduction

The skin fulfills a large range of functions, including the prevention of percutaneous water loss, temperature maintenance, sensory perception, and immune surveillance [1]. Moreover, skin health and appearance play crucial roles in self-esteem and social interactions [2].

Environment factors, such as sun exposure, smoking, and air pollution, as well as intrinsic factors like advancing physiological processes and poor nutrition, lead to skin aging, resulting in gradual dermal atrophy, fine and coarse wrinkles, and dry skin with loss of laxity, elasticity, and rough texture. Both the extrinsic and intrinsic factors contribute to skin aging with photoaging, i.e. long exposure to UV radiation is the primary factor of extrinsic skin aging [3-4].

Aging reduces the basal layer cell proliferation and leads to thinning of the epidermis, resulting in the reduction of the contact area between the epidermis and dermis, which reduces the nutrition of the epidermis. The inadequate nutrition weakens the ability of cells of the basal layer to proliferate [5-6]. In addition, the dermis of photo-protected aged skin shows not only fewer mast cells and fibroblasts than photo-protected young skin but also rarefied collagen fibers and elastic fibers [7].

Photoaging accounts for about 80% of facial aging [8], but it also causes epidermis thickening, which is in contrast to intrinsic factors, i.e. thinning of the epidermis leading to skin aging [9].

PRP has received extensive attention in recent years in different conditions like tissue regeneration, wound healing, scar revision, and alopecia, including skin rejuvenation [10]. The effects reported by the use of PRP are due to the presence of growth factors present in the platelets, which are released on activation.

Growth factors are derived from a subject’s whole blood. The blood is spun down in a centrifuge, which allows the red blood cells to be removed [11]. Most systems require a second centrifuging step to create the final product. Once the red blood cells are removed, the remaining plasma is again centrifuged, which allows the platelet-rich layer to be extracted, which is then activated with thrombin or calcium chloride. This activation step causes the platelets to begin releasing growth factors like vascular endothelial growth factor (VEGF) and fibroblast growth factor-2 (FGF-2), which enhances revascularization and angiogenesis, whereas collagen synthesis is believed to be stimulated by transforming growth factor-beta (TGF-β). Other growth factors like epidermal growth factor (EGF), insulin-like growth factor (IGF), and platelet-derived growth factor (PDGF) are also released.

PRP treatment achieved a faster wound healing rate in rabbits [12] compared to normal saline using Wockhardt’s Growth Factor Concentrate (Wockhardt Ltd., Mumbai, India) in excised wound model in diabetic and nondiabetic rats, which demonstrated better healing and contraction of the wound [13]. Significant improvement was observed in soft tissue healing in oral surgery by autologous PRP application [14]. The ability of autologous platelet gel (APG) to facilitate the proliferation of endothelial cells was confirmed by in vitro experiments [15]. PRP is expected to have a positive effect on facial rejuvenation and anti-aging due to its ability to facilitate collagen production, fibroblasts proliferation, and hyaluronic acid generation to increase dermal elasticity and keratinocyte proliferation [16-17].

Most of the PRP used for skin rejuvenation or anti-aging is injectable. Most of the PRP treatments on the face for rejuvenation are actually subdermal injections and very few topical PRP with GFC applications have been studied. Topical GFs can cross the skin barrier and bind to cell surface receptors, which trigger a signaling cascade and stimulate keratinocyte proliferation.

To obtain the maximum benefit from growth factors, it is usually thought that platelets should be maximally concentrated; however, if WBCs are simultaneously concentrated in the platelet fraction, the positive effects of growth factors may be reduced. Several studies demonstrate that the presence of WBCs and RBCs are detrimental to the healing effects demonstrated by the released growth factors. Most commercially available PRP preparation kits do not remove RBCs and WBCs and therefore do not harness the use of an acelluar growth factor solution that has several advantages. The Wockhardt process derives acellular growth factors from the subject’s own blood.

Wockhardt’s Autologous Growth Factor Concentrate (AGFC), MYOWNN™, is a topical application of essentially plasma that has been processed to contain a high concentration of platelets and growth factors but has an advantage over traditional PRP in that it is acellular and growth factor rich, i.e. without red blood cells (RBCs) and neutrophils which may cause pain and inflammation post-treatment. This study was designed to assess the safety and efficacy of a topically applied face serum - MYOWNN™ for anti-aging and facial rejuvenation.

## Materials and methods

This study was conducted in accordance with globally accepted standards of Good Clinical Practice (GCP) (as defined in the ICH E6 Guideline for GCP), in agreement with the Declaration of Helsinki, and in keeping with local regulations. Ethics committee approvals were obtained from Wockhardt Hospitals Institutional Review Board (ECR/624/Inst/MH/2014/RR-17) and Cutis Institutional Ethics Committee (ECR/930/Inst/KA/2017/RR-20).

In the format of an open-label study, 50 subjects (26 female and 24 male) seeking facial skin rejuvenation with Fitzpatrick skin type III-V [18] who have not taken any oral or topical treatments for at least four weeks before and any PRP-based treatment (injections) at least six months before the screening visit were recruited from two centers in India. Exclusion criteria were active acne, platelet count less than 150,000 µl, known history of bleeding disorders or hemoglobinopathies, or history of systemic disease resulting in an immunocompromised state affecting the ability to heal soft tissue. Complete details of the study procedure were presented to all the subjects and written consent was obtained.

For subjects who met the eligibility criteria and agreed to participate in the study, the facial assessment was done using Visage-LS (dermaindia®, Tamil Nadu, India) and a face analysis system; blood was collected for AGFC serum preparation. Demographic information, vital signs, and laboratory investigation were done to evaluate eligibility and safety during the study. Before application of the serum, subjects were instructed to wash their face, and if dryness was felt on the face, they were allowed to apply a moisturizer at least 20 minutes before the application of AGFC serum. However, in the case of oily skin, the use of a moisturizer was not recommended. Subjects were asked to apply a sunscreen lotion (at least 30 SPF) every three to four hours during the daytime for the entire study period. Subjects were instructed to avoid all kinds of cosmetic products, spa visits, any other facial treatment, and excessive exposure to sunlight during the study.

Blood collected for AGFC preparation was sent to Wockhardt’s processing facility in break-proof packaging. Blood vacutainers were centrifuged to separate platelets and then activated by the Wockhardt proprietary activator to get growth factor concentrate (GFC), which was extracted from the blood. Extracted GFC was transferred aseptically into sterile glass containers, which were then loaded in the lyophilizer. The lyophilization of GFC involved three basic steps, i.e., freezing, primary drying (sublimation), and secondary drying. Lyophilized GFC was then reconstituted with carrier serum and the Wockhardt proprietary permeabilizing agent to get the finished product. The finished product was shipped at 2-8°C to the study sites for a further dispensation to the subjects.

AGFC serum was dispensed to the study subject for daily application. The subjects were instructed to do a local application on the face once daily at night, approximately 30 minutes before sleeping, preferably. The dispensed preparation was sufficient for topical application on the face once daily at bedtime for three months. At the end of 3 months, subjects were re-consented to evaluate their willingness to participate for additional 2 months i.e. till the end of five months. Out of 50 subjects, 36 agreed to participate for an additional two months and provided blood for AGFC preparation.

All these subjects were followed up on a monthly basis till Month 3 and subjects who agreed to a re-bleed were followed till Month 5. At each monthly visit, subjects were evaluated for adverse events, concomitant medications, general facial examination, vital examination, treatment compliance since the last visit, and a facial assessment by the face analysis system. Objective analysis was performed by Visage-LS, a face analysis system, which is an advanced live status skin detection equipment together with shooting, analyzing, and displaying functions. It adopts the RGB (Red Blue Green), ultraviolet (UV), and polarized light (PL 3) spectrums combined with artificial intelligence and image analysis technology. It gives the live status of spots, pores, moisture, textures, wrinkles, and pigmentation. Subjective analysis was performed by globally accepted and validated scales like physician’s global aesthetic improvement scale (PGAIS), subject’s global aesthetic improvement scale (SGAIS), subject satisfaction score (SSS), and wrinkle severity rating scale (WSRS). PGAIS and SGAIS were scored from 1 to 5, with 1 being much worse, 2 being worse, 3 being no change, 4 being improved, and 5 being much improved. WSRS was scored from 1 to 5, with 1 being absent, 2 being mild, 3 being moderate, 4 being severe, and 5 being extreme. SSS were scored from 1 to 6, with 1 being very dissatisfied, 2 being dissatisfied, 3 being somewhat dissatisfied, 4 being somewhat satisfied, 5 being satisfied, and 6 being very satisfied.

All statistical analysis was carried out using the SAS software, Version 9.4 (SAS Institute Inc., Cary, NC). The primary efficacy endpoint was computed as the average of six skin anti-aging parameters (spots, pores, wrinkles, texture, moisture, and pigmentation) measured by the face analysis system (Visage-LS). The secondary efficacy endpoints were the individual skin anti-aging parameters (spots, pores, wrinkles and texture, moisture, and pigmentation) from the face analysis system, PGAIS, SGAIS, SSS, and the photographic assessment (using the WSRS scale).

A mixed model for repeated measures was used for this purpose. The model had change from baseline as the dependent variable visit as a factor and baseline assessment result as a covariate. All primary and secondary efficacy endpoints were analyzed using modified Intent-to-Treat mITT populations.

## Results

Out of the 50 subjects who were enrolled, 26 were female and 24 were male. The patients’ average age was 40 years, ranging from 30 to 55 years with a standard deviation of 8.62 years. According to the Fitzpatrick scale, 14 subjects (28%) had type III skin, whilst 27 patients (54%) had type IV and nine patients (18%) had type V skin.

A total of five adverse events were reported during the study and none of these events were considered related to study treatment. These adverse events were erythema, hypothyroidism, coronavirus disease (COVID) infection, and two events of acne. There were no serious adverse events or treatment-related adverse events reported during the study, as well as no adverse events leading to study discontinuation.

The results were analyzed from 47 subjects for Month 3 and 33 subjects for Month 5 who met the criteria for the mITT population. The mITT population included subjects who used at least one dose of study medication and had at least one post-baseline efficacy assessment.

On statistical evaluation in the mITT population, there was a statistically significant percentage change in an average of six skin anti-aging parameters (spots, pores, wrinkles, texture, moisture, and pigmentation) from baseline to Month 5. The improvement in percentage change in an average of six skin anti-aging parameters started right from Month 3 (p-value of 0.1047) but significant improvement was seen by Month 5 (p-value of 0.0150) (Table 1).

**Table 1:** Percentage Change in Average of Six Skin Rejuvenation Parameters - mITT Population * Statistically significant mITT: modified Intent-to-Treat

Percentage Change from	N	Mean	SD	p-value
Baseline to Month 3 (Day 105)	47	4.9	20.5	0.1047
Baseline to Month 4 (Day 135)	32	5.4	21.18	0.1126
Baseline to Month 5 (Day 165)	33	7.2	17.52	0.0150^*^

A statistically significant percentage change in individual skin anti-aging parameters like moisture, texture, and wrinkles was also visible at Month 5 (with p-values of 0.0006, 0.0054, and 0.0007, respectively) (Table 2).

**Table 2:** Percentage Change in Individual Skin Anti-aging Parameters - mITT Population * Statistically significant mITT: modified Intent-to-Treat

Percentage Change from	Mean	SD	p-value
Moisture			
Baseline to Month 3 (Day 105)	5.2	20.02	0.0792
Baseline to Month 4 (Day 135)	8.4	20.01	0.0130^*^
Baseline to Month 5 (Day 165)	11.6	19.25	0.0006^*^
Texture			
Baseline to Month 3 (Day 105)	7.4	26.00	0.0564
Baseline to Month 4 (Day 135)	7.3	27.45	0.0729
Baseline to Month 5 (Day 165)	9.8	21.52	0.0054^*^
Wrinkle			
Baseline to Month 3 (Day 105)	9.6	31.05	0.0387^*^
Baseline to Month 4 (Day 135)	12.6	33.38	0.0188^*^
Baseline to Month 5 (Day 165)	17.5	28.78	0.0007^*^

**Figure 1:**
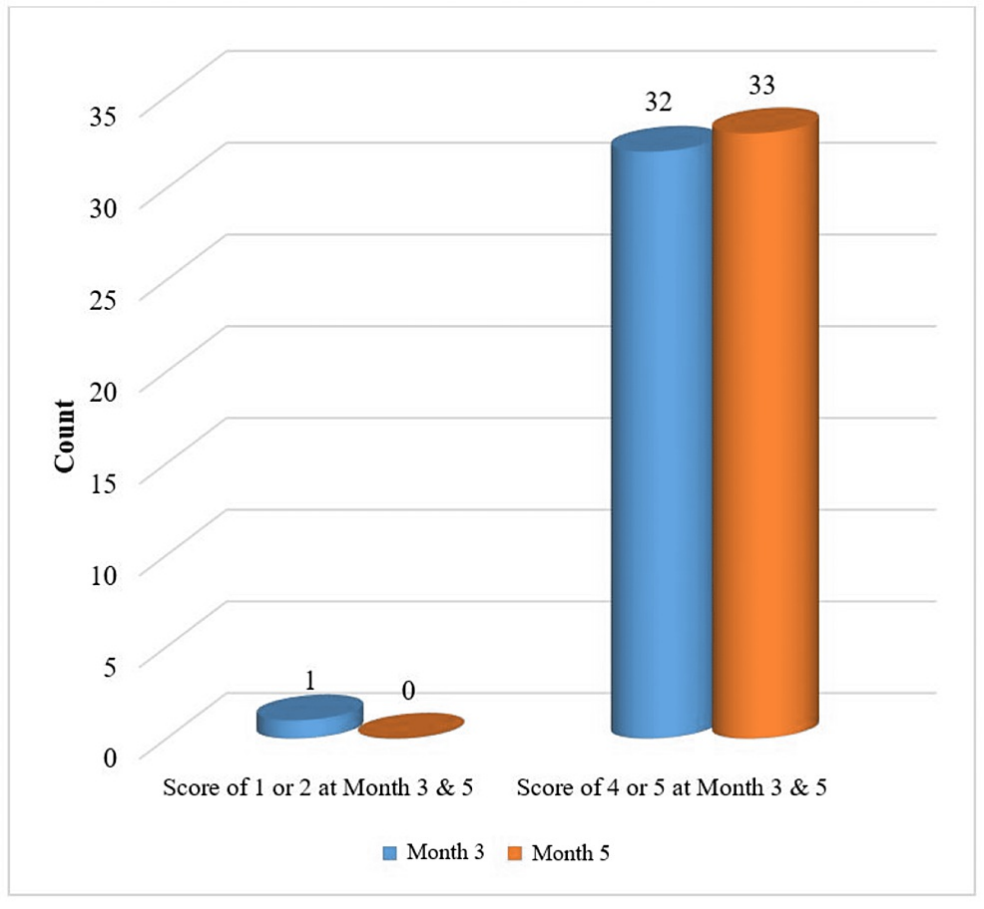
Improvement in Score of Physicians Global Aesthetic Improvement Scale (PGAIS) at Months 3 and 5 - mITT Population mITT: modified Intent-to-Treat

**Figure 2:**
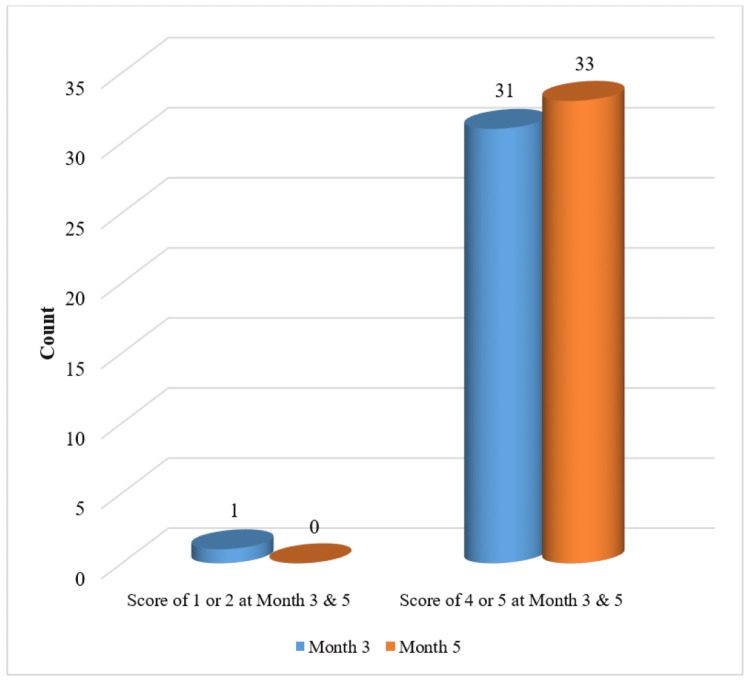
Improvement in Score of Subject's Global Aesthetic Improvement Scale (SGAIS) at Months 3 and 5 - mITT Population mITT: modified Intent-to-Treat

At the end of Month 5, the PGAIS maximum score of 4 or 5, i.e. improved or much improved, respectively, was reported in 100% of the cases (Figure 1), and on similar lines, the subjects also rated the SGAIS as the maximum score of 4 or 5, i.e. improved or much improved, respectively, in 100% of the cases (Figure 2).

**Figure 3:**
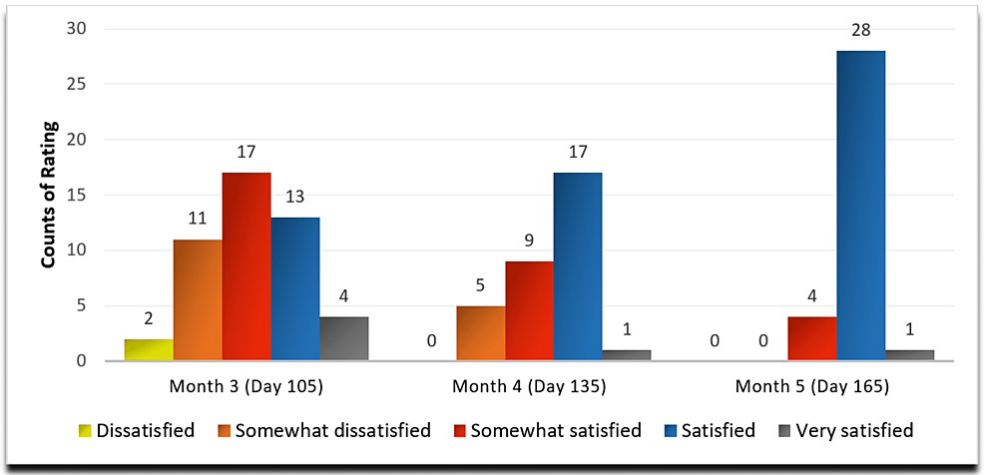
Subject Satisfaction Score (SSS) - mITT Population mITT: modified Intent-to-Treat

The subjects were asked to rate their satisfaction with SSS, whereupon at the end of Month 5, it was found that 12.12% of subjects were somewhat satisfied with the treatment, 84.85% subjects were satisfied, 3.03% subjects were very satisfied with the treatment, and none of the subjects were dissatisfied (Figure 3).

**Figure 4:**
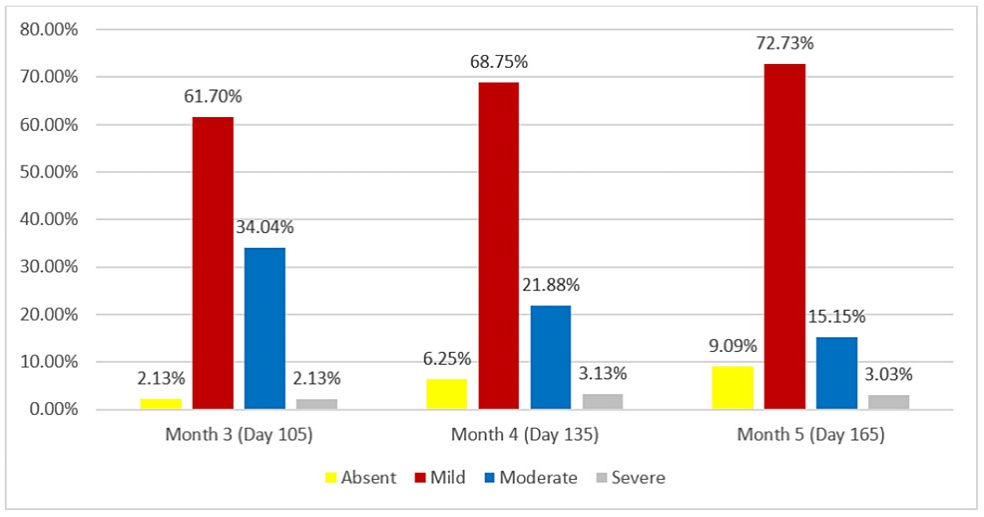
Wrinkle Severity Rating Scale (WSRS) - mITT Population mITT: modified Intent-to-Treat

**Figure 5:**
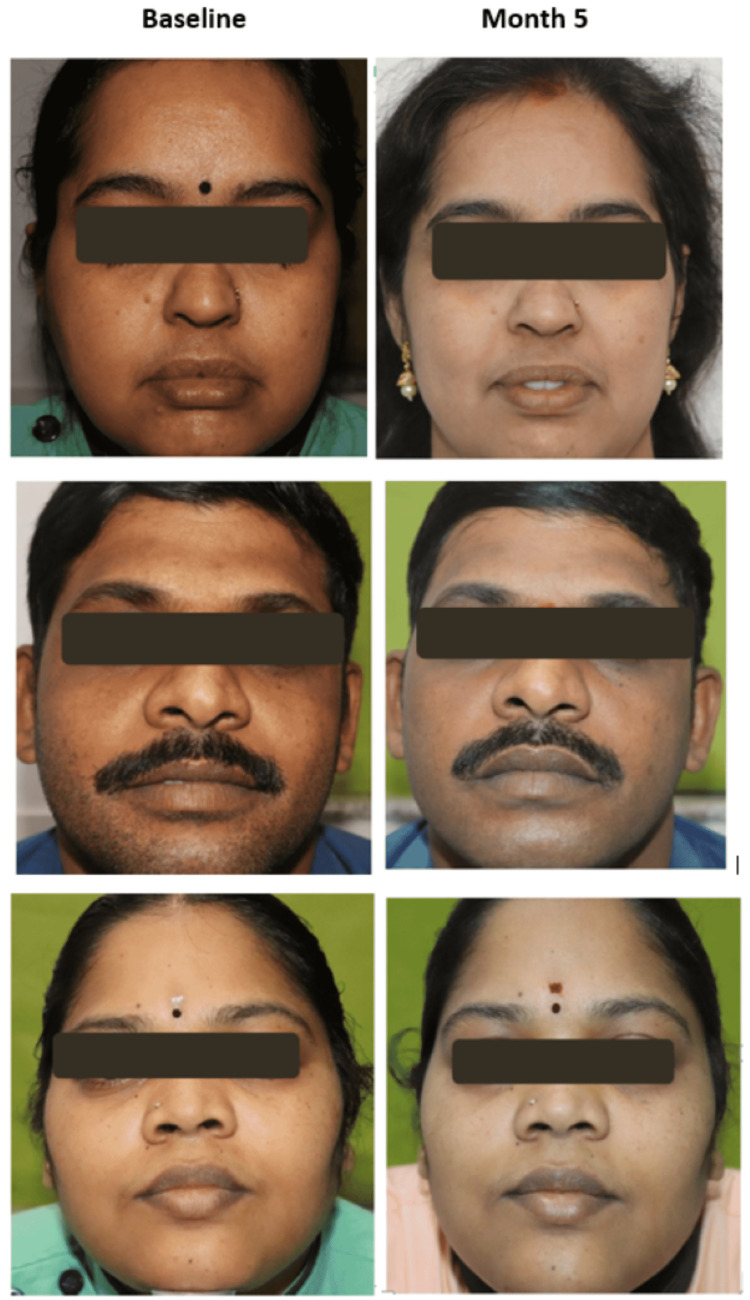
Photographic improvement from baseline to the end of Month 5

On an assessment of WSRS, it was found that at least one point improvement in wrinkles was seen in 14.89% of cases at the end of Month 3, which improved to 37.5% and 48.48% at the end of Months 4 and 5. respectively (Table 3, Figure 4). Photographic improvement from baseline to the end of Month 5 is depicted in Figure 5.

**Table 3:** Wrinkle Severity Rating Scale (WSRS) - mITT Population mITT: modified Intent-to-Treat

Score	Category	Description	Month 3 (Day 105)	Month 4 (Day 135)	Month 5 (Day 165)
1	Absent	Fold not visible; continuous line of skin	1 (2.13%)	2 (6.25%)	3 (9.09%)
2	Mild	Superficial fold, but visible and with a mild depression	29 (61.70%)	22 (68.75%)	24 (72.73%)
3	Moderate	Moderately deep fold, less than 1 mm deep	15 (34.04%)	7 (21.88%)	5 (15.15%)
4	Severe	Very long and deep fold, less than 2 mm deep	1 (2.13%)	1 (3.13%)	1 (3.03%)

Sensitivity analysis was also done to assess the efficacy parameters for the skin anti-aging parameters in subjects with age more than 50 years in the mITT population. The median percentage change from baseline to Month 3 (Day 105) was 4.1 compared to the median change in the overall population of 3.5, whereas the median percentage change from baseline to Month 5 (Day 165) was 7.3 compared to the median change seen in the overall population of 5.0. For the individual parameters like texture and wrinkles similar to the overall population and the trend in the median percentage change from baseline to Month 3 (Day 105) and Month 5 (Day 165) was seen. A statistically significant percentage change was seen in wrinkle parameters at Month 5 (p-value=0.0274) even with the small size (N=6) of this age population. For PGAIS and SGAIS assessment, at Month 5 (Day 165), all patients (6 (100.0%) of 6 patients) reported scores of 4 and 5. As per SSS, all patients were satisfied with the treatment at Month 3 (Day 105) and Month 5 (Day 165). For WSRS, at Month 5, five (83%) of six patients had scores between 1 and 3 and one (16.67%) of six patients had a score of 4; none of the subjects reported a score of 5.

## Discussion

Environmental factors and aging damage our skin and self-rejuvenation of the skin is supported by intrinsic growth factors that are generated by our own cells. As compared to young skin, aging skin generates fewer growth factors, which deems it necessary for the regular use of skin-care products with growth factors with the advancement of age basically to reduce the appearance of aging parameters like wrinkles, texture, fine lines, and tone [19].

The effects of three PRP injections over the course of 12 weeks were assessed on infraorbital wrinkles and skin tone in Asian subjects in a split-face study; this study showed significant improvement in both wrinkles and skin tone in infraorbital skin [20]. Dermaroller application of PRP over the subject’s malar area and forehead along with PRP injection into the wrinkles of crow’s feet biweekly for three times showed a statistically significant difference in skin firmness, wrinkles, and general appearance according to the grading scale of the patients before and after three PRP applications, whereas per the dermatologists, a statistically significant difference was seen only in skin firmness-sagging [17].

A limited number of controlled studies have been conducted to demonstrate that topically applied GFs can stimulate collagen synthesis and epidermal thickening, which is associated with clinical improvement in signs of photoaging. Also, very few studies have used objective analysis face systems to demonstrate improvement with the use of AGFC.

In this study, both an objective analysis with a face analysis system like Visage-LS, as well as a subjective analysis with different globally accepted scales like PGAIS, SGAIS, SSS, and WSRS was performed with topical application of AGFC.

In this open-label study, daily local application of AGFC over the face for three or five months showed a significant improvement in the average of six skin anti-aging parameters (spots, pores, wrinkles, texture, moisture, and pigmentation) from baseline. The improvement in percentage change in the average of six skin anti-aging parameters started right from Month 3 but significant improvement was seen by Month 5.

A statistically significant percentage change in individual skin anti-aging parameters like moisture, texture, and wrinkles was also seen. There was a statistically significant improvement in skin texture at the end of five months of application with a p-value of 0.0054, whereas a similar statistically significant improvement was seen in moisture as early as the end of four months of application with a p-value of 0.0130, which further improved by the end of five months of application with a p-value of 0.0006. The fastest and earliest improvement in individual skin anti-aging parameters was seen in skin wrinkles; a statistically significant improvement in wrinkles was seen as early as the end of three months of application with a p-value of 0.0387, which was further improved by the end of four months of application with a p-value of 0.0188, whereas the most improvement in wrinkles was seen by the end of five months of application with a p-value of 0.0007. Similar improvement was seen in subjects with age more than 50 years, especially in individual parameters like texture and wrinkles.

Our study results are in line with other studies that have used injectable PRP, and our study also used an objective advanced face analysis system to assess the difference.

There were no serious adverse events or treatment-related adverse events reported during the study as well as no adverse events leading to study discontinuation. There were no clinically meaningful laboratory-related changes observed and no clinically significant changes in vital signs parameters were observed during the study.

Our study had a few limitations like the relatively small sample size. Also, this was a non-comparative study but comparative studies are difficult to design in this indication, as variability in inter-individual skin conditions and skin aging factors is very high and can confound the results.

## Conclusions

Improvements in the average of six skin anti-aging parameters, as well individual parameters like wrinkles, moisture, and texture, depicted by objective analysis with a face analysis system were complemented by subjective analysis performed by different globally accepted scales like PGAIS, SGAIS, SSS, and WSRS. There were no safety concerns seen with the use of MYOWNN™ in this study, and long-term safety is being evaluated in an ongoing study.

In conclusion, this study showed that topical MYOWNN™ treatment started showing a difference as early as three months with significant improvement by Month 5, and MYOWNN™ can be safely and effectively used for anti-aging and face rejuvenation.
